# Client Perspectives of Family Therapy: A Qualitative Systematic Review

**DOI:** 10.1111/jmft.70024

**Published:** 2025-04-30

**Authors:** Elif Todd, Rachael Pond, Kitt Coomber

**Affiliations:** ^1^ Private Practice Palmerston North New Zealand; ^2^ Development Institute of Education Massey University Palmerston North New Zealand; ^3^ Head of Guidance Awatapu College Palmerston North New Zealand

**Keywords:** client experiences, client views, family counseling, family therapy, qualitative, systematic review

## Abstract

This qualitative systematic review aimed to synthesize and discuss family members' perspectives of helpful and unhelpful factors in family therapy (FT) sessions, to benefit the application of FT interventions and training. Eleven studies met eligibility criteria and were critically appraised and thematically synthesized. Four themes resulted: therapist qualities contributing to the therapeutic alliance, practitioners' use of therapeutic techniques, intervention delivery, and family engagement with the process. Helpful factors included therapist warmth, kindness, and genuine care; therapist connecting with family in a sensitive, respectful, and nonjudgmental manner; effective use of therapeutic techniques that facilitated self‐reflection, emotional expression, communication, and perspective‐taking; therapy sessions conducted collaboratively with active family participation; focusing on family strengths and resources; and tailoring format to family needs. Unhelpful factors included participants sharing before ready, therapist siding with a family member, therapy process not matching family needs, and insufficient progress early on. Implications for practice and future research are discussed.

## 1 Introduction

Family therapy (FT) addresses challenging emotional, psychological, behavioral, and relational dynamics. It is effective for anxiety and mood disorders, behavioral problems, attachment problems, relationship issues, recovery from child abuse and neglect, substance abuse, somatic problems, first‐episode psychosis, and schizophrenia (Carr 2009, 2019; Lebow 2020). Family theorists assert that the source of distress in the identified client (whose challenges brought family members to FT) is relational, caused by problematic interactional patterns (Reiter 2018; Walrond‐Skinner 2014). FT aims to create systemic changes that lead to positive outcomes and well‐being for all individuals in the system (Gurman and Kniskern 2014; Lebow 2018).

With service users reporting that over half their reasons for engaging in therapy are family conflicts, FT approaches have become among the most widely practiced modalities in the mental health field worldwide today (Hecker and Wetchler 2015; Phipps 2019). Cook et al. (2010) found that family systems therapy was the second most practiced model after cognitive behavioral therapy, with 49% of practitioners using it, pointing to the incorporation of systems thinking and practicing of family interventions by a broad range of mental health professionals. Early schools and theories of FT have evolved into numerous approaches, including Strategic Family Therapy, Bowen Family Systems Therapy, humanistic and experiential approaches such as Satir's communication model and Johnson's Emotionally Focused Family Therapy, and postmodern approaches such as narrative and solution‐focused family therapies (Borsca and Stratton 2016; Gehart 2018).

### 1.1 Family Therapy Research

Research into the effectiveness of FT has grown since the 1980s (Gladding 2015; Kaslow 2011). As the emphasis on empirically supported treatments increased, comprehensive, widely disseminated integrative family interventions and manualized approaches, such as Functional Family Therapy (FFT), were developed (Heatherington et al. 2015). These integrative, manualized FT approaches address specific client populations and presenting issues such as adolescent conduct disorder, behavioral and emotional challenges, substance abuse, and eating disorders (Kaslow 2000; Pote et al. 2003) and are some of the most widely researched family interventions (Asen 2002; Sexton et al. 2003).

For example, there is much evidence that FFT improves adolescent externalizing behavior and reduces family conflict (Hartnett et al. 2017; Robbins et al. 2016) and that Multisystemic Therapy (MST) decreases antisocial behavior and improves parenting skills, family relationships, and social support (Henggeler and Schaeffer 2016; Sawyer and Borduin 2011). There is also considerable research on the effectiveness of Brief Strategic Family Therapy in changing patterns of family interactions that allow problematic adolescent behaviors (Szapocznik et al. 2012) and of Multidimensional Family Therapy in reducing aggression, crime, and substance use and improving mental health and school and family functioning (van der Pol et al. 2017). In Aotearoa/New Zealand, where the authors are based, MST was found to significantly reduce offending and improve family functioning and school attendance (Russell 2008). Pae Whakatupuranga, an adapted version of FFT that incorporates te ao Māori and Pasifika frameworks and therapeutic approaches, was also deemed effective for both Māori and non‐Māori families (Heywood and Fergusson 2016). Conversely, there is research that counters the efficacy of these manualized models and points to concerns of rigidity (Davies 2019; Littell et al. 2021) and researcher bias (Weisman and Montgomery 2019).

Whilst some interventions have been extensively researched for efficacy, other FT approaches such as psychodynamic FT, object relations therapy, and narrative FT have a limited research base in comparison, reducing their likelihood of appearing in empirically supported treatment lists (Katafiasz et al. 2019; Larner 2004). This has resulted in a division between well‐researched and under‐researched interventions and concern that some therapies are becoming less practiced while others are establishing themselves as preferred modalities around the globe (Heatherington et al. 2015; Lebow 2020).

Further, most FT research is quantitative and outcome‐based, examining efficacy (how FT can be done right), effectiveness (how FT can be done well), and efficiency (how FT can be done quickly and economically) (Baucom and Crenshaw 2019; Sexton and Datchi 2014). There are many meta‐analyses and reviews, for example, Goger and Weersing (2022), Peris et al. (2021), and Stith et al. (2022), and mixed‐methods reviews combining quantitative and qualitative data, for example, Byrne et al. (2020) and Helps and Le Coyte Grinney (2021), that point to favorable outcomes of FT for various challenges such as anxiety, suicidal ideation, eating disorders, substance use disorders, abusive behaviors, and schizophrenia, and for various formats of FT, such as telehealth and web‐based delivery.

Although FT research has progressed to incorporating cognitive, cultural, and emotional perspectives of individuals, these perspectives are typically studied quantitatively through scales and inventories, for example, Pinsof et al. (2008) and Tilden and Wampold (2017). Qualitative process research that aims to produce “practice‐based evidence” about how FT works is mostly through observational methods or those that analyze in‐session therapy discourse and change processes, for example, Lebensohn‐Chialvo et al. (2019) and Ray et al. (2019). While there are studies on self‐reported client experiences of FT, most of these focus on outcomes rather than in‐session experiences, for example, Hopkins et al. (2017) and O'Neill (2017). Currently, two systematic reviews of qualitative research have been published about FT. One synthesized client experiences of a broad range of conjoint therapy sessions, including couples and multifamily sessions (Chenail et al. 2012). Its findings support common factors research on what makes therapy successful in general, as well as couple and FT‐specific common factors. It further discusses the element of a within‐family alliance. The other systematic review specifically examined client experiences of reflecting teams in various clinical settings (Harris and Crossley 2021).

### 1.2 Research Rationale, Aim, and Review Questions

Considering how prevalent FT is, it is important for the field to hear and learn from the voices of family members as the active partners of FT interventions. Understanding client experiences of what is useful or not during sessions is particularly important for family therapists, FT and counseling educators, and FT students. With a gap of over a decade since the publication of Chenail et al.'s (2012) systematic review, it is time for renewed exploration. The purpose of this systematic review is, therefore, to provide a contemporary and comprehensive overview of client perspectives on what is helpful and what is not helpful during FT sessions. It further aims to help FT practitioners to identify their strengths and needs for development. Its findings may also be helpful for practitioners of individual therapy who might wish to include family members in the therapeutic process. The review question specifically asks what family members experience as helpful and unhelpful during FT sessions. This review does not describe and differentiate between different types of FT as its focus is client experiences of FT in general.

### 1.3 Personal Positions of the Authors

The authors, who include practicing counselors (E.T. and K.C.) and an academic (R.P.), share a commitment to understanding how individuals and families experience and make sense of therapeutic processes. Grounded in systemic and relational approaches, we recognize the importance of amplifying the voices of young people, adults, and families rather than imposing professional assumptions. Our collective expertise spans individual, couple, and family counseling, school‐based counseling, human development, and qualitative systematic review methodology, allowing us to bridge theory and practice. We value research that enhances the responsiveness, inclusivity, and relational depth of therapy, ultimately strengthening therapeutic support for diverse individuals and families.

## 2 Methods

We conducted a systematic review because systematic reviews identify, appraise, and combine data from all relevant studies that attempt to answer a specific question, thus increasing the generalizability and transferability of results (Boland et al. 2017). They also limit researcher bias by following an explicit set of structured steps (Liberati et al. 2009).

The epistemological lens that guides this review is rooted in social constructivism, which posits that reality and knowledge are constructed through experience and social interaction (Barbosa da Silva 2008; Waller et al. 2016). People understand and construct meaning related to a specific phenomenon through their experience and reflection (Adom et al. 2016). Adopting this perspective, the review aims to explore the subjective FT experiences and perceptions of participants in qualitative studies. Qualitative research explores diverse perspectives on complex and nuanced phenomena and provides in‐depth, rich, and detailed information on contextual factors, subjective meanings, and processes (Creswell 2013).

Qualitative systematic reviews have a distinct advantage in exploring the depth and richness of subjective human experiences of the phenomenon being studied, developing enhanced understanding through the synthesis of individual primary studies (Flemming and Noyes 2021). We therefore employed a qualitative systematic methodology to identify, analyze, and synthesize findings from qualitative studies that captured lived experiences, perceptions, and interpretations of FT participants to understand both helpful and unhelpful aspects of FT from the perspective of those experiencing it. The findings of qualitative systematic reviews can further help conceptualize theoretical frameworks and interventions, inform policies and practice guidelines, and guide further research (Saini and Shlonsky 2012). This is another aim of this review.

### 2.1 Inclusion and Exclusion Criteria

The present systematic review included qualitative studies on any FT approach; however, eligible studies needed to explore experiences or perspectives of recent FT (within approximately 2 years of data collection to minimize recall bias) or ongoing FT and involve at least two family members attending the majority of FT sessions together, with interviews conducted either separately or jointly. Data collection from one participant warranted inclusion. Eligible studies also needed to be published between January 1, 2011 and December 31, 2021 in a peer‐reviewed journal (to ensure reliability of findings and adherence to ethical standards). Due to limitations in time and resources, only studies published in English were eligible. Studies needed to be published after the eligibility date of Chenail et al.'s (2012) systematic review to prevent duplication and fulfill their recommendation that this study area be further explored.

As the review sought participant experiences during sessions, we excluded phenomena such as referrals, help‐seeking, or outcomes. Further exclusions were inpatient/residential, school and community, parent education, and psychoeducation programs; support groups; research participants under 6 years old as these children generally require different qualitative interviewing methods (Platt 2016); specific interventions or practices not generalizable to most FT settings (e.g., use of one‐way mirrors, teams of therapists, and reflecting teams); family interventions for eating disorders (e.g., where the family component focused on symptom reduction through parental responsibilities, such as meal planning); individual or couples therapy where FT models were applied; and FT where family members consistently attended separately.

### 2.2 Search Strategy

The first author searched the EBSCO‐Discovery Service (including PsychInfo and Web of Science) and Scopus databases extensively and systematically in May 2022 with the guidance of two university librarians. In coordination with the third author, the first author revised and applied the search strategy again in June and September 2022. Search terms, reported in Table 1, were based on the SPIDER framework (Cooke et al. 2012) and used in multiple combinations. The first author also searched the tables of contents of relevant FT journals and hand‐searched the reference lists of pertinent articles to identify any studies that may have been missed in the database searches.

**Table 1:** SPIDER search terms.

Sample	client*, participant*, member*, famil*, child*, parent*, patient*, “young person*”, caregiver*, consumer*, “service user*”, adolescen*, teen*
Phenomenon of interest	attitude*, experience*, perspective*, perception*, feeling*, evaluation*, view*, thought*, voice*
Intervention	“family intervention*”, “family therap*”, “family counsel*”, “family psychotherap*”, “system* therap*”, “family therapy technique*”, “family therapy activit*”, “family therapy tool*”
Design	qualitative, phenomenolog*, interview*, “thematic analys*”, “grounded theor*”
Evaluation	satisfaction, engagement, interaction*, participation, refusal, dropout, evaluation, assessment, preference*, barrier*, persever*
Research type	*qualitative*

### 2.3 Critical Appraisal

To critically appraise the overall quality and methodological rigor of the selected studies, the first author used a modified version of the Critical Appraisal Skills Program (CASP) checklist (Long et al. 2020). This is a widely used tool, which provides structured checklists for assessing the quality and trustworthiness of qualitative evidence synthesis in health and social sciences. It includes 10 questions to help identify limitations that might affect review quality. In coordination with the second author, the first author evaluated each study using the modified CASP checklist, assessing criteria such as research goal and design, participant selection, appropriate methodology and data analysis, clarity and credibility of findings, potential bias, and other ethical considerations, and systematically recorded responses for each criterion and categorized studies based on their overall quality. This appraisal process informed the interpretation of findings by highlighting methodological strengths and limitations.

### 2.4 Data Extraction and Synthesis

The first author extracted and tabulated study characteristics (such as participant demographics, FT model, and data collection and analysis) and study findings; the second and third authors reviewed these. For the data synthesis, Thomas and Harden's (2008) thematic synthesis and Braun et al.'s (2019) reflexive thematic analysis were both selected; the former for its method of bringing together heterogeneous qualitative studies (Lockwood et al. 2017) and the latter for its clearly identified steps and emphasis on reflexivity (in this case through a detailed reflexive journal). Preferred Reporting Items for Systematic Reviews and Meta‐Analyses (PRISMA) guidelines were followed to maintain reporting standards. The first author carried out the synthesis and analysis of data and the second and third authors critically examined these processes. Peer researcher review and feedback were also sought and considered throughout. For the synthesis, the first author read and reread the articles to ensure familiarity with the data, and then, with the review questions in view, coded relevant participant quotes (first‐order constructs) and researcher interpretations (second‐order constructs) from the findings sections of each article. These were tabulated into adjacent columns to avoid anecdotalism and misrepresentation, as recommended by Popenoe et al. (2021). The analysis process was iterative, not linear. Initially, data were inductively coded. After coding several studies, the first author developed a codebook with a list of refined codes; this added a deductive coding element. The list evolved through the coding process as an understanding of and engagement with the data deepened (Braun and Clarke 2021). At least one code was given to each extract and marked as positive (helpful), negative (unhelpful), or neutral experiences. They were also marked as applying to parents/caregivers (henceforth referred to as “parents”) or to children/adolescents/young people (henceforth referred to as “young people”).

As coding progressed, several possible themes were developed; as such, coding and theme generation stages took place simultaneously. During this process, the first author made an effort to eliminate potential biases by coding all relevant data reflexively, with attention to any preconceived interpretations. As the analysis was carried out by one researcher, the coding and initial theme generation stages were conducted twice to ensure faithfulness to the data, as recommended by Popenoe et al. (2021). The first author then grouped codes into overarching categories, or initial themes and subthemes, some of which had been generated during the coding process. While most codes fit into these themes, there were several outlier codes. Once the initial themes and subthemes were reviewed and checked for accuracy and consistency with the data, they were labeled. The second author critically examined these themes and subthemes and minor adjustments were made.

### 2.5 Ethical Considerations

Since we did not collect any primary data, the review did not need approval by the Massey University Human Ethics Committee; we lodged a low‐risk notification instead. Nevertheless, we carefully considered ethical issues relevant to qualitative systematic reviews. To mitigate bias, we strove to remain open to broader interpretations that reflect the complexities of family experiences, making an effort to include and represent all research participants' views relevant to the research aims and to represent the work of primary researchers fairly and respectfully, remaining attentive to viewpoints that challenged our own beliefs. We revisited the findings of each study to ensure that participant experiences had not inadvertently been missed. We considered multiple points of view—those of the authors of the current review and other colleagues—to manage any personal positions and biases through regular individual and peer reviews, reflections, discussions, feedback, and reflexive journaling. At each stage, the first author engaged in continuous critical reflection on both theoretical and personal assumptions to avoid seeking confirmatory evidence in the data, while carefully considering how knowledge was being produced and whom it might benefit—or potentially harm.

## 3 Results

### 3.1 Study Selection

**Figure 1:**
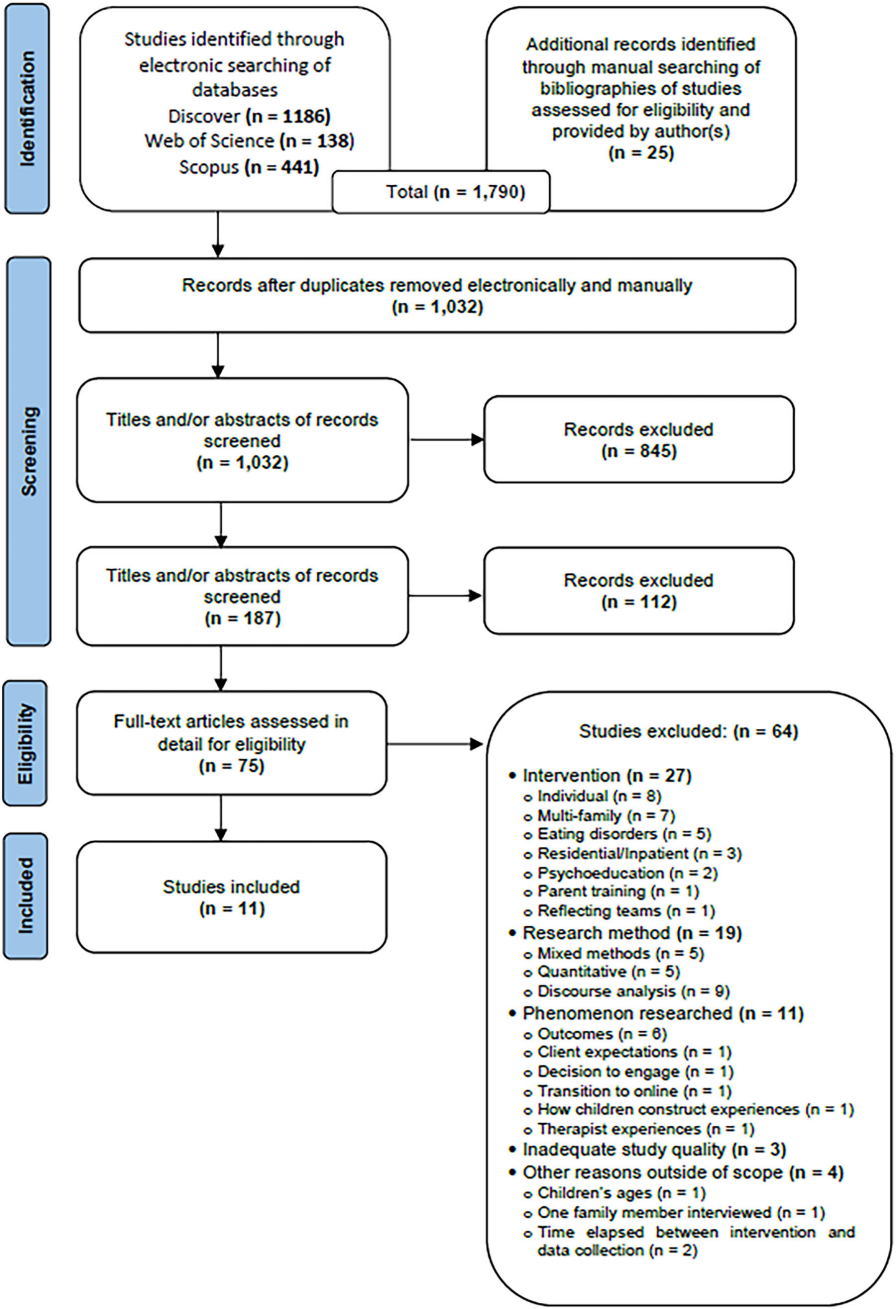
Flow diagram of the study selection process. [Color figure can be viewed at wileyonlinelibrary.com]

Figure 1 presents the selection process in a PRISMA flow diagram (Page et al. 2021). Database and hand‐searching identified 1790 studies with 1032 remaining after deduplication. The first author scanned the titles and abstracts of these and selected 75 relevant studies for full‐text screening. She contacted authors of potential studies to locate missing information and clarify uncertainties. An additional article published in 2021 was provided by the author of an identified study. In total, 64 full‐text studies were excluded for not meeting one or more inclusion/exclusion criteria (Figure 1 reports the main ones). Eleven articles were eligible and selected for the systematic review.

### 3.2 Study Characteristics

Table 2 shows the characteristics of the 11 studies. Of these, seven were from England and the remaining four were from Malta, China, Scotland, and the United States of America. Studies were heterogeneous in terms of FT approaches, population, and intervention duration and frequency. Two studies reported young people's perspectives only and two studies reported parents' perspectives only. Two sets of studies were related: Bunting et al. (2021) reported young people's perspectives and Fox et al. (2016) reported parent perspectives of the same intervention; similarly, Paradisopoulos et al. (2015) reported young people's perspectives and Kaur et al. (2017) reported parent perspectives of the same intervention. Gilson and Abela's (2021) research was the only case study with one participating family.

**Table 2:** Data extraction table showing a summary of study characteristics.

#	Study (authors, year, geographical location)	Participant demographics (number, age, gender, ethnicity/race/country of origin)		Study aim	Family therapy (FT) model	Data collection and analysis
		Children/Young people	Parents			
1	Bunting et al. (2021), England Related study: Fox et al. (S3)	*n* = 7 Age range: 14–18 Gender: *f* = 4, *m* = 3 Ethnicity/Race: Samoan, Kenyan/Sudanese, Georgian, Black British (*n* = 2), Moroccan, British		To explore minority ethnic young people's experiences of Multisystemic Therapy (MST), including aspects of the intervention, which facilitated or hindered engagement and change.	MST − Up to 3 times/week over a period of 3–5 months (20–60 sessions).^a^ − Individual, couple, and family sessions depending on family need.^a^	Grounded theory analysis of 40–90 min individual semistructured interviews 3–24 months after participants' clinical cases were closed (dropout or intervention completed).
2	Collyer et al. (2021), England	*n* = 10 Age range: 10–16 Gender: *f* = 3, *m* = 7 Ethnicity: White/White British (*n* = 8), Black/Black British (*n* = 2)	*n* = 19 Parent demographics not reported	To understand factors relating to dropout in Functional Family Therapy (FFT) by exploring the experience of families who have completed FFT and those who have dropped out from therapy.	FFT Average number of sessions: *n* = 10 families who completed all three phases of FFT over 3–6 months. *n* = 4 families who did not complete all three phases.	Thematic analysis of individual semistructured interviews post participation, either in person or over the phone.
3	Fox et al. (2016), England Related study: Bunting et al. (S1)		*n* = 7 ethnic minority completer caregivers Age: 40s–60s Gender: *f* = 6, *m* = 1 Country of origin: Rwanda, Jamaica, Israel, Ghana (*n* = 2), China, Ireland	To understand what contributed to or hindered engagement and change from the perspective of minority ethnic caregivers and relate this to the MST structures that guide assessment, treatment planning, and intervention.	MST − Up to 3 times/week over a period of 3–5 months (20–60 sessions).^a^ − Individual, couple, and family sessions depending on family need.^b^	Grounded theory analysis of 60–90 min semistructured interviews 0–12 months postintervention.
4	Gilson and Abela (2021), Malta	*n* = 3^c^ Age range: 7–14 Gender: *f* = 1, *m* = 2 Maltese family	*n* = 2 Mother (40s) Father (40s)	To explore the therapeutic alliance between the family therapist and the family who are experiencing a relational trauma.	Systemic FT. Individual, couple, and family sessions with some or all members of the family attending. Therapy had not concluded at the time of data collection.	Narrative case study. System for Observing Family Therapy Alliance, storying, and cross‐sectional analysis of brief interviews with the children (unclear whether individual or together) and one interview with the parents.
5	Kaur et al. (2017), England Related study: Paradisopoulos et al. (2015)		*n* = 12 caregivers Age: 30s–50s Gender: *f* = 11, *m* = 1 Ethnicity: White British (*n* = 11), Black British Caribbean (*n* = 1)	To investigate caregiver perspectives during and after therapy to determine how families achieve and sustain change through MST.	MST − Up to 3 times/week over a period of 3–5 months (20–60 sessions).^d^ − Individual, couple, and family sessions depending on family need.^d^	Grounded theory analysis of 45–81 min semistructured interviews 5–21 months postintervention.^e^
6	Liu et al. (2020), China	*n* = 12^f^ Age range: 16–18 Gender: *f* = 10, *m* = 2	*n* = 24 (*n* = 12 mothers, *n* = 12 fathers)	To explore the experiences of FT by depressed Chinese young people and their families.	Milan Systemic Family Therapy 10–11 ×90‐min sessions every 2–3 weeks over 5–6 months. Both parents attended FT with the adolescent.	Thematic analysis of approximately 90‐min semistructured family interviews postintervention.
7	McPherson et al. (2017), Scotland	*n* = 12 Age range: stratified 11–13 and 14–17 Gender: *f* = 6, *m* = 6	*n* = 14 (*n* = 11 mothers, *n* = 3 grandmothers)	To explore the views and experiences of families and practitioners on the implementation of FFT and identify key barriers and facilitators to successful implementation.	FFTParticipants had completed all three phases of FFT treatment over 3–6 months.	Thematic analysis of semistructured individual interviews 0–18 months postintervention.
8	Morino (2019), England	*n* = 4 Age range: 10–14 Gender: *f* = 2, *m* = 2 Ethnicity/Race: White = 3, Black = 1		To explore client and therapist perspectives on the change process during systemic psychotherapy for children with conduct disorder in the Child and Adolescent Mental Health Services (CAMHS) setting to generate ideas for further therapeutic work.	Home‐based psychotherapy is based on systems theory and various FT models, such as narrative, solution‐focused, structural, and Milan systemic therapies, incorporating parenting and social skills psychoeducation. Other details (e.g., number of sessions and session frequency) not provided.	Grounded theory analysis of 90‐min family or parent/caregiver interviews during midphase of therapy (session 6 or later).
9	Paradisopoulos et al. (2015), England Related study: Kaur et al. (2017)	*n* = 8 Age range: 13–18 Gender: *f* = 5, *m* = 3 Ethnicity/Race: White British (*n* = 5), Black British Caribbean (*n* = 1), Mixed ethnicity (*n* = 2)		To generate a theory of what contributed to sustained improvements for young people who attended MST as well as challenges they encountered and compare this to the existing literature on the process of change.	MST − Up to 3 times/week over a period of 3–5 months (20–60 sessions).^a^ − individual, couple, and family sessions depending on family needs.^a^	Grounded theory analysis of semistructured interviews 5–21 months postintervention.^e^
10	Thompson et al. (2011), United States of America	*n* = 38 (19 families with one young person and one caregiver in each family interviewed)		To examine the perceptions of troubled youth and their caregivers' use of family play and experiential activities during FT sessions.	Solution‐focused FT and experiential activities.^g^ Up to 12 × 60–90 min sessions.	Semistructured family interviews in families' homes approximately 3 months after the intervention. The interviews were audio‐recorded and transcribed.
		Age range: 12–16 Gender: *f* = 13, *m* = 6 Ethnicity/Race: Latino (*n* = 10), White (*n* = 6), Black (*n* = 3).	Mothers (*n* = 12), fathers (*n* = 3), grandmothers (*n* = 2), aunts (*n* = 1), uncles (*n* = 1). Age range: 29–63 Ethnicity: Latino (*n* = 10), White (*n* = 6), Black (*n* = 3)			
11	Tighe et al. (2012), England	*n* = 16 Age range: 13–17 Gender: *f* = 2, *m* = 14 Ethnicity/Race: *n* = 9 Black, *n* = 8 White, *n* = 3 mixed ethnicity, *n* = 1 Asian	*n* = 22 (*n* = 19 mothers, *n* = 1 father, *n* = 1 couple)	To investigate families' views of the strengths and limitations of MST's delivery model and what helped or hindered positive change.	MST Average number of sessions: 33. Range of number of sessions: 7–68. Therapy duration: 23 weeks. Range of therapy duration: 13–36 weeks.	Thematic analysis of semistructured individual interviews (60 min for parents, 30 min for young persons (range: 19–43) approximately 2 months postintervention.

^a^ *Information source:* Secondary researcher via email correspondence.

^b^ *Information source:* Primary researcher via email correspondence.

^c^ This was a four‐child family, but one child's responses were excluded due to the age being outside the inclusion criteria.

^d^ *Information source:* Tertiary researcher via email correspondence.

^e^ Only families who had achieved positive changes (no new convictions, in education or training, and still living at home) at the end of their completed MST intervention (as defined by the three MST ultimate outcomes) were interviewed.

^f^ Participants were all one‐child Chinese families where the adolescent had been diagnosed with major depressive disorder, first‐episode depression with no remission.

^g^ At the beginning of each family session, an activity that involved experiential interactions and skill‐building exercises was conducted. Topics of these activities focused on problem areas the family had identified, such as anger management, problem solving, improving communication, substance use, expressing feelings, coping, and so forth.

As Table 2 shows, over half of the studies used a manualized FT intervention: five used MST and two used FFT. In three studies the model was Systemic Family Therapy (with one incorporating several other therapy models), and in one study it was Solution‐Focused Family Therapy. A total of 210 family members contributed to the selected studies: 119 parents (69 female, 22 male, and 28 gender not reported) and 91 children (46 girls and 45 boys). Where reported, parents' ages ranged from 29 to 69 years. Children's ages ranged from 7 to 18 years. In all studies, the identified client was a young person.

### 3.3 Quality of the Studies

As shown in Table 3, three studies were of high quality, meeting all assessment items of the modified CASP checklist (Long et al. 2020). Five were of moderate quality, and three were of lower quality. Studies were assessed as moderate or low quality for not sufficiently justifying the recruitment strategy; only collecting and reporting one‐sided data (what was helpful/positive); not sufficiently describing the data analysis process; and not discussing findings in sufficient detail. Further, several studies did not adequately discuss the relationship between the researcher(s) and participants; thus, whether they considered the influence of their role and/or potential bias was not clear. Two did not mention ethical considerations; however, it is assumed they followed ethical requirements as they were published in peer‐reviewed journals. Despite variations in quality, all studies made important contributions and were deemed valuable.

**Table 3:** Quality assessment of studies using the modified Critical Appraisal Skills Program.

Checklist question	1. Bunting et al. (2021)	2. Collyer et al. (2021)	3. Fox et al. (2016 )	4. Gilson and Abela (2021)	5. Kaur et al. (2017)	6. Liu et al. (2020)	7. McPherson et al. (2017)	8. Morino (2019)	9. Paradisopoulos et al. (2015)	10. Thompson et al. (2011)	11. Tighe et al. (2012)
1. Was there a clear statement of the research?	✓	✓	✓	✓	✓	✓	✓	✓	✓	✓	✓
2. Is the qualitative methodology appropriate?	✓	✓	✓	✓	✓	✓	✓	✓	✓	✓	✓
3. Was the research design appropriate to address the aims of the research?	✓	✓	✓	✓	✓	✓	✓	✓	✓	✓	✓
4. Was the recruitment strategy appropriate to the aims of the research?	✓	✓	S	✓	✓	✓	✓	✓	✓	✓	✓
5. Was the data collected in a way that addressed the research issue?	✓	✓	U	✓	✓	✓	✓	✓	U	✓	✓
6. Was the relationship between the researcher and participants adequately considered?	✗	S	✗	S	S	✓	✓	✓	✗	✓	S
7. Have ethical issues been taken into consideration?	✓	✓	✗	✓	✓	✓	✓	✓	✗	✓	✓
8. Was the data analysis sufficiently rigorous?	U	✓	✓	✓	✓	✓	U	✓	✓	✓	✓
9. Is there a clear statement of findings?	✓	✓	S	✓	✓	✓	✓	✓	✓	✓	✓
10. How valuable is the research?	V	V	V	V	V	V	V	V	V	V	V
Overall quality	★	★★	★	★★	★★	★★★	★★	★★★	★	★★★	★★

*Notes:* ✓ = yes (item adequately addressed); ✗ = no (item not adequately addressed); S = somewhat; U = unclear (insufficient information).

For Question 10: V = valuable; S = somewhat valuable.

Overall quality: ★ = low; ★★ = moderate; ★★★ = high.

### 3.4 Data Synthesis

**Figure 2:**
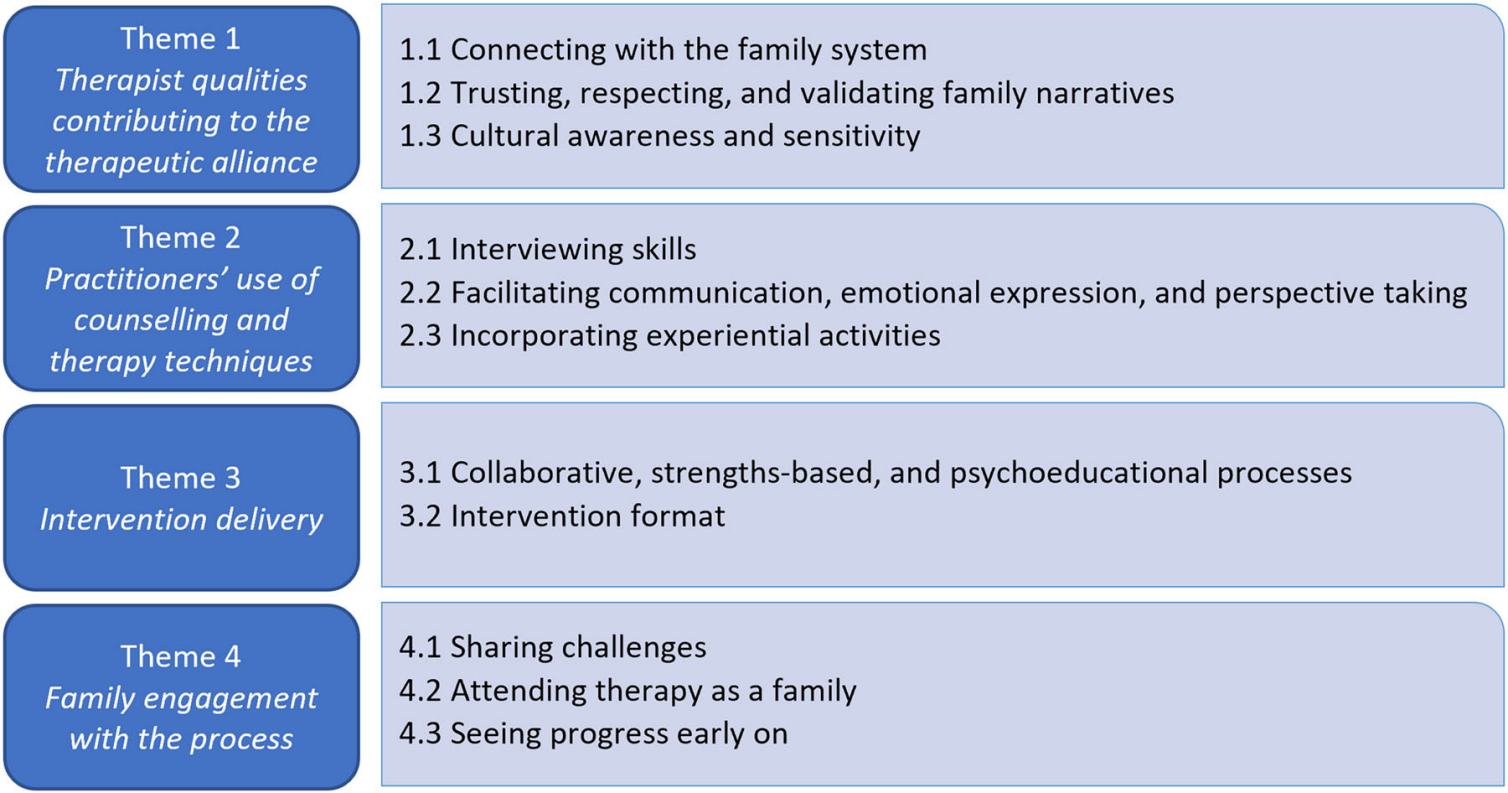
Themes and subthemes. [Color figure can be viewed at wileyonlinelibrary.com]

As Table 4 shows, despite the heterogeneity of modalities and delivery formats, similarities in many key findings were evident. Four broad themes were identified: therapist qualities contributing to the therapeutic alliance (TA), practitioners' use of counseling and therapy techniques, intervention delivery, and family engagement with the process. Themes and associated subthemes are shown in Figure 2 and presented below with illustrative participant quotes. All themes and subthemes applied to both young people and parents; thus, the words “families” and “participants” are used interchangeably when reporting participant views. When a particular view is applied to a specific group only, this is indicated. Participant quotes are denoted as “YP” for the young person and “P” for the parent.

**Table 4:** Data extraction table showing summary of findings.

#	Study	Findings (domains/categories/themes‐subthemes)	
1	Bunting et al. (2021)	*Understanding family culture* Understanding, respecting, and being knowledgeable about culture Addressing different views on wanting outside help *Considering cultural differences within the family* Acknowledging the role of cultural/acculturation differences in the young person's presenting difficulties Being culturally sensitive and facilitating perspective‐taking Differences in discipline practices	*Understanding the young person's unique cultural values/beliefs* Viewing their identity as a cultural blend Therapist exploring identity and enabling sharing this with parents/caregivers Therapist understanding, respecting, and being knowledgeable about culture *Recognizing and reflecting on cultural differences in the therapeutic relationship* Therapists addressing their own ethnicity and cultural identity
2	Collyer et al. (2021)	*Relational engagement* Problem definition Balance of alliance Openness	*Perceptions of helpfulness* Credibility Relevance *Practical barriers* Convenience and accessibility
3	Fox et al. (2016)	*Creating a shared position* Therapist “joining/aligning” with family Having shared goals *Developing a therapeutic alliance* Valued therapist qualities Developing a positive working relationship *Considering cultural difference* Being culturally understood Therapist taking a culturally sensitive approach Therapist understanding and respecting difference Considering the role of culture in difficulties *Working within a safe and trusting relationship* Establishing a trusting relationship with the therapist Being supported to risk trying new things Working collaboratively	*Empowering the parent/caregiver* Taking a strengths‐based approach Focusing on successes Therapist helping to manage the power differential within and outside the family *Increased communication within and outside of the family* Increasing positive communication between parents/caregivers and young person Increasing positive communication between the family and other agencies *Therapist acting as a cultural broker* Facilitating perspective taking Negotiating cultural differences in the family Being helped to contextualize young people's behavior in UK culture Therapist acting as a cultural reference point
4	Gilson and Abela (2021)	*The “Way of Being” of the therapist is key to engagement* The ability to be intuitive The ability to be self‑reflexive The ability to keep maneuverability in the dialogical space The ability to hold neutrality *Consolidating alliances by creating conjoint and separate spaces*	*A creative play‑based approach helps in building alliances* *Balancing the benefits and drawbacks of involving children* *Making therapy meaningful for all* *Showing compassion toward parents* *Navigating an alliance with the school and the mental health service*
5	Kaur et al. (2017)	*Transforming the relationship to help* From ambivalence to trust Facilitative Multisystemic Therapy (MST) therapist qualities and approach Renewed possibilities to seek future help *Caregiver‐therapist alliance as a helpful model* Safety in the relationship facilitating risk‐taking Experiencing a collaborative relationship *Therapist supporting a family alliance* Facilitating connections between the family Valuing family goals	*Privileging a positive story* Accepting differences Respecting independence Recognizing exceptions & strengths in my child *Increased positive communication in relationships* Increased communication with the school Maintaining open expressive communication within the family *Sharing responsibility for change* Recognizing each other's roles Balancing my input
6	Liu et al. (2020)	*Factors supporting therapeutic alliance formation* The therapist's personality traits The therapist's professionalism Similarity between the therapist and the clients *The therapist's systemic attitudes* Nonpathological perspective Nonauthoritative manner Therapists' unconditional respect and acceptance	*Systemic therapy direction* Promoting adolescents' individual development Facilitating emotional expression and flow *Effective systemic therapy techniques* *Strategies for dealing with current issues* Dealing with crisis issues Coping with academic difficulties
7	McPherson et al. (2017)	*Engagement of families* Trust and honesty Seeing the need and experiencing change Views of significant others Psychosocial and cultural influences	*Structure and delivery* Flexibility is key Work and homework can be fun Home‐delivery
8	Morino (2019)	*Good therapeutic relationship* Having someone to talk to Being acknowledged and legitimized Active communication and therapy style: good relationship with children *Good access* Being offered help when needed Accessible language Being aware of one's own ability	*Resources* Confidence Knowledge, advice, information, and practical help Asking for help *The sense of agency versus being hopeless, having no choice, having no say about one's own matters* Change is caused by one's own action versus change as beyond one's control Being able to control the situation versus inability to control the situation Coherent story of success versus a fragmented story of success Time: children grow out of bad behavior
9	Paradisopoulos et al. (2015)	*The therapeutic alliance: A model of safe and trusting relationships* Creating a context of safety, trust, and collaboration Revising expectations of MST and the therapist Having choice and control in MST: developing a sense of personal agency *Improving self and other awareness and recognizing responsibility* Taking personal responsibility for change Recognizing the impact of behavior on self and others *Increasing systemic awareness* Increased reflexivity and perspective‐taking Identifying unhelpful patterns of interaction and increasing positive communication Raising other's awareness and understanding	*Acknowledging progress and celebrating success* Noticing positive changes in self and family Being realistic about the level of change *Having alternatives: the continued contribution of strategies and techniques* Having strategies to control emotions Creating structure and routine *Identifying and planning for a preferred future* Recognizing and pursuing passions: forming an identity Leaving the past behind Determination and perseverance Working on being a family
10	Thompson et al. (2011)	*Positive responses to activities* Sharing openly Fun/Entertaining Application to daily lives Enhanced family interactions Leading to insight and awareness	*Negative responses to activities* Some activities unproductive Superficial atmosphere, especially when the activity did not fit the family mood Not enough advice and guidance from a therapist Some activity rules were difficult to follow and created complexity Discomfort about the emotional content brought up by the activities The same issues were discussed despite a variety of activities Point of some activities is unclear
11	Tighe et al. (2012)	*At the family's convenience* In the family home Fitting around the family's schedule *Holistic approach: Working with systems around the young person* Working with parents/caregivers to impact the young person Working with systems beyond the home (school, extended family, other agencies) Promoting prosocial activities and relationships *Solution‐focused, practical approach, providing observable benefits* Seeing change early on Practical help, hands‐on support Psychoeducation: Advice, guidance, and fresh ideas Applying behavioral parenting strategies: Contracts, rewards, and consequences	*Strong therapeutic relationship: A person‐centered, collaborative approach* The therapist is able to connect with the family—down‐to‐earth, easy to talk to Empathy, understanding, and genuine care Nonjudgmental and nonblaming Collaborative—working together as equals *Therapist as a source of support: Companion, counselor, motivator, mediator* Parent/caregiver has someone there for them—companion Someone to talk to—counselor Encouragement to persist in implementing strategies—motivator Facilitating communication and understanding—mediator Confident, authoritative parenting—feels stronger, more in control

Table 5 shows the contribution of studies to the themes and subthemes.

**Table 5:** Contribution of the studies to the synthesis.

Themes and subthemes	Bunting	Collyer	Fox	Gilson	Kaur	Liu	McPherson	Morino	Paradisopulos	Thompson	Tighe
Theme 1: Therapist qualities contributing to the therapeutic alliance	✓	✓	✓	✓	✓	✓	✓	✓	✓		✓
Connecting with the family system	✓	✓	✓	✓	✓	✓	✓	✓	✓		✓
Trusting, respecting, and validating family narratives	✓	✓	✓	✓	✓	✓	✓	✓	✓		✓
Cultural awareness and sensitivity	✓		✓			✓					
Theme 2: Practitioners' use of counseling and therapy techniques	✓	✓	✓	✓		✓	✓	✓	✓	✓	✓
Interviewing skills		✓	✓	✓		✓	✓	✓	✓		
Facilitating communication, emotional expression, and perspective‐taking	✓	✓	✓	✓		✓			✓		✓
Incorporating experiential activities				✓		✓	✓			✓	
Theme 3: Intervention delivery		✓	✓	✓	✓	✓	✓	✓	✓		✓
Collaborative, strength‐based, and psychoeducational processes		✓	✓	✓	✓	✓	✓	✓	✓		✓
Intervention format		✓		✓	✓	✓	✓	✓			✓
Theme 4: Family engagement with the process	✓	✓		✓			✓	✓	✓		✓
Sharing challenges		✓		✓				✓	✓		✓
Attending therapy as a family	✓	✓		✓							
Seeing progress early on		✓					✓	✓			✓

## 4 Theme 1: Therapist Qualities Contributing to the TA^1^

As is evident from Table 5, this was the most prevalent theme with 10 contributing studies. A safe and supportive therapeutic relationship was identified by families as an essential element of effective therapy, a transformative factor in continuing therapy. Therapist qualities and characteristics were identified as the main factors influencing the strength of this bond.

### 4.1 Connecting with the Family System

In all but one study,^10^ families reported that the way their therapist engaged with them had a significant effect on the therapeutic process. Therapist characteristics such as warmth, kindness, empathy, sincerity, reliability, and a sense of humor helped families feel at ease and trusting of the process. A young person stated, “The FFT practitioner treated us as people rather than a job … she was nice and warm and friendly.”^7^ This in turn influenced how comfortable and open they were in sessions. Families appreciated therapists who wanted to get to know them and cared for their well‐being and positive outcomes. For one parent, this was what motivated her to do the work: “And because she cared that much I had to give it 100%.”^11^ Therapists offering support outside of sessions, especially in crisis situations, led to families feeling cared for and supported. For example, a father was comforted when the therapist's response to his daughter's disclosure of having thoughts of dying was, “… I will try to stop you because I care about your safety. If you have any more impulses to hurt yourself in the future [contact me].”^6^ Further, families valued therapists' ability to connect with all family members in this way, creating “simultaneous alliances” with each family member and honoring these alliances, even during sessions where not all family members were present.

### 4.2 Trusting, Respecting, and Validating Family Narratives

Participants in these 10 studies cited how feeling understood and valued provided confidence in the therapeutic process and hope for the future. They felt validated when therapists acknowledged their experiences rather than considering them as abnormal, imagined, or pathological.

My parents have been trying to comfort me, telling me not to worry, as if my fears were wrong. That makes me feel embarrassed and guilty. However, [the therapist] told me, ‘You can worry; we can do something else to let go of that worry,’ and it made me feel comfortable; it's like he was closer to my feelings.^6^

(YP)

The importance of this nonjudgmental, nonpathologising view is also applied to the family system, helping families approach the problem as a systemic issue.

We are a family, we have different perspectives. She did not target him as the problem, she considered the whole family.^4^

(P)

Further, families valued therapists who saw them as part of a wider system (e.g., extended family and school), considered those in the process, and liaised with and linked families to external support systems. Families valued this “ecological systems approach” as it helped to dispel the view of the family as a pathological system. When families' stories were trusted and respected, confidence in their capacity to make and maintain changes increased. Further, respect for their worldview, values, emotions, and expectations of therapy provided participants with a sense of empowerment and enabled them to try new ways of being. Many participants described recreating this relationship with their family members. Conversely, there were some participants who felt misunderstood: “I felt like she did miss our points quite a lot. She was looking at a tiny, tiny bit of it not the overall sort of thing”^2^ (YP). Additionally, younger children expressed a wish to be more meaningfully involved.^4^

### 4.3 Cultural Awareness and Sensitivity

As shown in Table 5, three studies reported families' appreciation of therapists who understood, valued, and respected their culture, helping create a positive attitude towards the process.

She did respect my culture … That was important. If she had not gone through that line, I wouldn't have worked with her.^3^

(P)

Families valued therapists who viewed the family culture in relation to their own culture and discussed possible meanings of cultural differences at the onset of therapy. This led to increased trust in the therapist and the process. There were parents who valued being able to consult the therapist about cultural and age‐appropriate behavior within this trusting relationship. Some participants preferred a therapist from a similar culture or background, saying that this would help them feel better understood.

Cause she was from a similar background, she wasn't being biased … she was understanding of both my parents and me … I think a [White] British person would have understood less.^1^

(YP)

Further, some parents said that they would be more open to being challenged by a therapist from a similar culture. While some young people voiced concern that a therapist from a similar culture might be manipulated by their parents, others said that a therapist from a different culture would not necessarily succeed as a cultural mediator, expressing concern that their parents might not understand, or refuse to accept, that cultural differences played a part in problems. Lastly, a few participants expressed neutrality, saying that what mattered was therapist training and approach, specifically their consideration and self‐education of the client's culture.

## 5 Theme 2: Practitioners' Use of Counseling and Therapy Techniques

This was another prevalent theme, with contributions from 10 studies, as Table 5 shows. It refers to the therapeutic skills and techniques that enable family members to communicate in new ways and at deeper levels, understand themselves and each other better, and see their challenges in different ways.

### 5.1 Interviewing Skills

As shown in Table 5, families in seven studies appreciated practitioners who listened actively, paraphrased, reflected, checked for accuracy, gave all family members equal “time and space,” and used age‐appropriate, down‐to‐earth, jargon‐free language. A young person stated, “She gives a chance to everybody … When I speak she listens and she does not listen to anyone else.”^4^ Therapists' calm speaking style positively influenced young people's listening skills. Their effective questions led to deep reflections and insights, drawing participants into the process and helping them make links to unresolved issues. For example, a mother said, “She has even asked me questions that have helped me reflect on past experiences. Today I realize that this is probably why I am tired, as all my life I have had to take care of others.”^4^ Effective questions also helped participants recognize their own knowledge and strengths, prompting them to devise their own solutions.

He's very, very clever at that. And it was very annoying at one point because I would ask for his advice and I was answering myself [laughter]. But it made me understand that if I look hard enough I can find the answers …^8^

(P)

One study^3^ mentioned the importance of practitioners refraining from making assumptions and instead checking client perspectives and meanings.

### 5.2 Facilitating Communication, Emotional Expression, and Perspective‐Taking

Families in seven studies (see Table 5) identified practitioners' skills in objectively helping family members convey their thoughts and emotions to each other, creating a significant shift in the emotional flow within sessions.

I start speaking and she listens, then we reason it out together, then she changes what I said into her own words and she says it in a way that my parents can understand it.^4^

(YP)

Some participants observed *circular questioning*, a strategic FT technique that helps family members see from multiple viewpoints. This facilitated perspective‐taking, enabling family members to consider multiple points of view. As one young person stated:

[The therapist] asked a lot of interesting questions. He asked my father ‘Could you guess what your son thinks of your relationship with your wife,’ or ‘What kind of suggestions do you think your son would like you to give him?’ These questions were very enlightening and helped us think from the perspectives of the others.^6^

Participants generally expressed therapist objectivity when facilitating perspective‐taking; however, some family members believed that therapists tended to side with the young person and place blame on the parent, negatively affecting their credibility. Some participants observed practitioners using *reframing*, a therapy technique that enabled families to view their challenges from a different lens. By assisting families to reframe their stories, practitioners enabled parents to reassess the narrative around the young person to be less problem‐saturated, assigning positive re‐interpretations and meanings to behaviors that were previously deemed problematic and unacceptable.

Don't take the idea that her ‘brain can't work’ as an obstacle … see what positive aspects it can bring. It seems that her depression can bring a temporary calm to our family.^6^

(P)

Similarly, young people saw their parents' behaviors differently. For example, reframing enabled young people to reinterpret what they had deemed controlling behaviors as attempts at connection and protection. This skill also led to insights about own behaviors, helping family members to move toward accepting differences and needs and developing family patterns that promoted boundary awareness and individual growth and differentiation. In bicultural families, reframing facilitated conversations about cultural influences on needs and behaviors. Reframing “problem” behaviors helped family members understand each other's attempts to navigate cultural contexts.

### 5.3 Incorporating Experiential Activities

Four studies (see Table 5) contributed to this subtheme to varying degrees. The study^10^ that focused solely on family perspectives of experiential activities used during FT made the largest contribution. The activities included drawing, clay sculptures, visualization exercises, family and community maps and diagrams, and interactive games. Families described experiential activities as fun and entertaining, an enjoyable way to start sessions, increase energy and spontaneity, enable relaxing and opening up, and encourage positive feelings and participation. Activities created opportunities for family members to enjoy each other; they welcomed these positive interactions as opposed to problem‐saturated conversations.

Participants commented that activities enabled them to share thoughts and emotions they would not have during talk‐oriented sessions. They found themselves discussing important issues without setting out to do so: “… we were actually doing something else and then it all came out during the middle of it”^10^ (YP). Talking about family issues as part of a game prevented anger and blow‐ups, enabling family members to interact in a respectful, calm manner, which helped neutralize problems. When problems were not personalized, families were able to approach them constructively.

[The activity] almost puts the problem in the middle … it's not where we're looking at it and going ‘you, your problem’ [pointing finger], it's not specifically you … the problem is more in the middle.^10^

(P)

Skill‐building activities on communication and conflict resolution helped family members practice new ways of being with each other. Some participants reported holding onto activity materials and objects (e.g., worry boxes) as visual, physical, or metaphorical reminders and motivators for these newly developed skills.

Some participants described experiencing deeper emotional levels during activities. These emotionally profound therapeutic experiences led to an increased understanding of self and others and helped increase empathy and motivation to establish new interactions and new ways of being with each other. In contrast to the usual conflictual dynamics at home, these activities helped them see positive aspects and characteristics in themselves and their family members, leading to perspective changes. Although most family members described experiential activities as valuable, some found them unproductive and artificial. Further, when they did not fit the family's mood or need, when rules were complex, or when debriefing took too long, activities became irrelevant and nontherapeutic. While some young people expressed discomfort about the emotional content the activities elicited, they were also seen as an integral part of the therapeutic process.

## 6 Theme 3: Intervention Delivery

As shown in Table 5, 10 studies contributed to this theme which incorporates family perspectives of how FT was conducted: for example, the setting, the content, and the process (regardless of the FT model).

### 6.1 Collaborative, Strength‐Based, and Psychoeducational Processes

Families in nine studies (see Table 5) appreciated an approach that saw them as active contributors and knowledgeable, equal partners in a process where family strengths and resources were affirmed. Further, families valued information, strategies, and resources provided by therapists; these gave them confidence and options, assisting therapeutic progress.

First, a collaborative approach where families and practitioners worked together in identifying needs, setting goals, and deciding on the treatment content, methods, pace, and solution options gave participants confidence in their role in the process and a sense of control over their challenges. When this collaborative element was absent and the focus of the therapeutic work was not in keeping with family needs and goals, participants viewed the intervention as being irrelevant and lost confidence in the possibility of change.

… it wasn't practical solutions, it was things that we just thought well it's not really practical … it was more of a perfect family solution, and that's obviously not what we are, which is why we went.^2^

(P)

Second, families highly valued an approach that reinforced what was working and focused on positive patterns and strengths. This perspective instilled hope and empowerment. When therapists were motivating and encouraging, parents felt hopeful that things could improve and were more inclined to persevere. For example, a mother said that she could take control of her situation because she was “… put back in charge by the therapist and made aware of [her] own ability to be in control ….”^8^ Another parent said,

She kept feeding me my strengths … I was feeling I was just shit but she was saying, you know, “Look, think about what you've done.” … She praises you … I felt held, I felt supported, I felt encouraged.^11^

Lastly, participants appreciated practitioners who shared their professional knowledge and expertise about the family challenges with honesty and openness. Therapists leading open discussions about mental health and providing psychoeducation on symptoms increased family members' understanding and provided relief from anxiety caused by uncertainties. Participants further appreciated the provision of skills and tools to assist in communicating, identifying triggers, regulating emotions, and managing conflicts outside of sessions.

It was just the things I was getting told to do like go upstairs, calm down, like write my feelings down and that.^9^

(YP)

A particular strategy, behavioral contracting, where consistent rules, rewards, and consequences are established to help eliminate emotional tension from conflicts, was identified as a factor in creating change, especially when the children were younger. Families with older adolescents said that even though mutual understanding was more effective than creating contracts, they “… allowed you to begin the process of negotiation and agreement … that for me was really something quite significant*”* ^5^ (P). Some young people said that although their initial reactions to behavioral contracts were negative, they appreciated the clarity and structure they created.

### 6.2 Intervention Format

As shown in Table 5, seven studies mentioned participants' views of the therapy format, including where sessions took place and how therapists responded to families' requirements, for example, some individual sessions. A parent stated, “I think there's certain things that (my son) probably would have said without me being there.”^2^ While most clients of home‐based interventions mentioned this as a factor in continuing sessions, some young people found the home environment distracting from the content: *“*You don't concentrate as much in my house. If it was in a library I would concentrate more.”^7^ Several families referred to the limitations of the intervention format with regard to the frequency of sessions. One study^1^ discussed families' perspectives on the end of therapy: Although most parents thought therapy ended at the right time, some found the ending too early or too abrupt; they described a tapered ending where some check‐in sessions and/or the possibility of contacting the therapist for advice as being preferable. Lastly, several participants said that they would have liked some individual sessions to support and complement family sessions.

## 7 Theme 4: Family Engagement With the Process

As Table 5 presents, six studies contributed to this theme which comprised participant views on “opening up” in a therapeutic environment, individual versus family sessions, and early signs of progress.

### 7.1 Sharing Challenges

Five studies (see Table 5) reported participant views on opportunities to talk about their worries and challenges, describing a sense of release and unburdening through expression, described by one parent as “… offloading some of my bits onto somebody else.”^11^ Another mother shared this about her young child who had been complaining about ongoing stomach pains:

… just after he had spoken up about what annoys him at home, he came and told me in my ear, “the pain has subsided.”^4^

While opening up and engaging in difficult conversations often led to insight and awareness, some participants reported feeling discomfort and anxiety about opening up. They further expressed concern that they might be judged or blamed if they openly discussed their challenges, particularly their parenting.

I think you get frightened you're gonna be told you're a bad mum or you've done something wrong.^2^

(P)

This discomfort often eased after the initial sessions, after the therapeutic relationship had been built.

### 7.2 Attending Therapy as a Family

Several participants reported a preference for therapy that addressed the family system as opposed to focusing on an individual.^1,2,4^ Despite at times being more complex and uncomfortable, family sessions were a significant factor in therapeutic progress.

It's one way listening to the sound of one instrument, and another listening to a symphony. She has taken a holistic approach.^4^

(P)

Parents valued the awareness that family sessions created of their part in the problem and what they could change, citing the ineffectiveness of previous (individual) therapy that cut them off from the therapeutic process.^4^ Several participants further reported that the father's presence, or lack of it, had a significant impact. A mother said that the father's engagement filled her with hope and courage.^4^ A young person, who did not complete the intervention, said, “I think my dad should have attended, 100%. It would have been good to work with my dad.”^2^

Some participants reported that attending as a family was important because it enabled them to hear their family members' accounts. This helped them develop understanding and empathy and gain insight into the effects of their own behaviors on the family system.

I think I just like just stopped and just thought about all the things that I'd done and how did my mum take it when I was listening to my mum talk to [the therapist] and start crying and stuff. I didn't really know that it did like hurt my family so much.^11^

(YP)

Conversely, some young people^2,11^ felt discomfort and did not engage with the therapist or the process, during either family or individual sessions.

I ain't a strong communicator anyway, that's one of my problems … I have anxiety issues as well and she just made me feel really uncomfortable.^2^

(YP)

Further, one study^4^ reported that younger children felt distressed during whole‐family sessions (one young child to the extent of dissociating), specifically when family members argued and they were unable to challenge inaccurate accounts.

### 7.3 Seeing Progress Early On

As shown in Table 5, four studies reported that signs of early progress and positive changes such as better communication, spending time together as a family, and improved parenting skills increased engagement and motivation to continue FT.

Just every session it was getting better and it helped ….^7^

(YP)

In contrast, families experienced disappointment and disengagement when the focus was not relevant to family needs, the purpose and potential benefits were not clear, and/or they did not see progress early on.

## 8 Discussion

The aim of this systematic review was to explore client perspectives on what is helpful and not helpful during FT sessions. Eleven qualitative studies provided data for analysis. Data were synthesized into four overarching themes: therapist qualities contributing to the TA, practitioners' use of counseling and therapy techniques, intervention delivery, and family engagement with the process. The findings and their implications are discussed below.

### 8.1 Therapist Qualities Contributing to the TA

The most cited helpful component was the TA, which supports prior research (Sprenkle et al. 2013; Wampold and Imel 2015). First, the therapist's qualities and characteristics, their *way of being*, had a direct impact on this relationship, in keeping with existing literature (D'aniello and Fife 2020). Existing research also supports the correlation between client feelings of safety and trust and therapist characteristics, such as kindness, empathy, and genuine care for the family's well‐being and therapy outcomes (Sackett and Cook 2022).

Second, when therapists expressed nonjudgmental, respecting, and validating views of the family's experiences, participants felt relieved, motivated, and hopeful. Families found it helpful when practitioners viewed the family system as the client rather than targeting the “identified client” for change. Moreover, positioning the family as part of a series of systems currently not functioning well was helpful in dispelling the view of a “problematic family,” as supported by existing literature (W. Y. Lee 2020).

Third, therapists' respect for their clients' values, lifestyles, and worldviews impacted the TA. Although few studies contributed to findings on ethnic, racial, and geographical cultural factors, some participants from dual‐cultural contexts trusted a therapist from the same or similar ethnic background more, expecting to be understood better. Some young people were concerned that a therapist of a similar race or ethnicity displayed bias and/or was ineffective in navigating cultural mediation. Despite these preferences and concerns, what mattered most to participants was for therapists to practice cultural competency and sensitivity, supporting previous research (Friedlander et al. 2011).

These findings point to several significant implications for practitioners and educators. First, as a strong therapeutic relationship is seen to have a major impact on the client's view of a positive therapeutic process and the therapist's characteristics and qualities directly impact this relationship, it is important that practitioner education programs emphasize interpersonal processes and incorporate research findings on the self‐of‐the‐therapist (Sprenkle et al. 2009) element into therapist training and practice. The therapist's way of being (how they regard their clients and how they interact with them) not only directly affects the quality of the therapeutic relationship but also their effective use of therapy techniques, regardless of the model (Davis and Hsieh 2019; Holyoak et al. 2021). Thus, it is crucial for therapists to practice self‐reflection and self‐awareness and engage in continuous personal and professional development and supervision.

Second, as FT means multiple relationships in the room, it is necessary for therapists to create simultaneous alliances and show the same care and respect for all individuals as well as the family system. This has been previously reported (Friedlander et al. 2011) and further supports Chenail et al.'s (2012) findings. Moreover, it is important to honor multiple alliances even when not all parts of the system are present (Sprenkle et al. 2013).

Third, it is important for practitioners to learn about the family culture, value cultural differences, and consider the complexities of cultural loyalties and impositions (D'Aniello et al. 2016). As cultural competency encompasses all cultural aspects of families and individuals within them, it is imperative for practitioners to develop themselves in this regard to prevent any biases from shadowing the TA (Pakes and Roy‐Chowdhury 2007).

### 8.2 Practitioners' Use of Counseling and Therapy Techniques

Findings point to the significance of effective use of therapeutic techniques and skills. Attentive and empathic listening and questions that led to reflections, awareness, and insights were identified as helpful. Families described *reframing*, which provided them with alternative views of their challenges, as a helpful technique. *Circular questioning*, which therapists used to facilitate perspective‐taking, was also described as beneficial. As previous research has also found, it is not enough that therapists hold and validate multiple viewpoints; what enables insight and change is the skill of enabling other family members to see and hold these also (Chenail et al. 2012).

Although the terminology for the techniques mentioned was only used by primary researchers and not by research participants, families noticed their nature and usefulness. This raises the question of whether other well‐known FT techniques such as reenactments, unbalancing, tracking, ordeals, or genograms were used. Although originating in specific models, these useful FT techniques are often employed in multiple approaches (E. Lee et al. 2018; W. P. Russell and Breunlin 2019). A recommendation for practitioners and educators would be to aim for competency in learning and practicing these techniques.

Some participants cited instances where the therapist's efforts to facilitate perspective‐taking did not result in the desired outcomes, particularly when the discussions were about cultural aspects of the family's culture and that of the “host country.” Families in contributing studies reported that when therapists sided with a family member, often the young person, others felt blamed and became defensive; this negatively affected the TA and the therapist's credibility. A significant implication for practitioners is to be aware of the potential risk of bias toward an individual in the system, leading to loss of trust. Practitioners must ensure that support for the young person does not present as blame of parent(s) (Friedlander et al. 2011), avoid triangulation within sessions, and notice and repair any relationship ruptures, providing balanced and fair avenues for expression (Chenail et al. 2012; Helimäki et al. 2022a).

The current review also found that experiential activities that shifted the focus from the personal to the family system were helpful. Various fun and interesting tasks that got family members communicating and collaborating led to positive interactions and emotional expression. Although at times the emotional content brought up created discomfort, this was deemed necessary. Activities were found to be unhelpful when they did not fit the family's needs or mood, seemed superficial, had complex rules, or took too long. Physical reminders of activities (e.g., family personality profiles, stones representing life stressors, etc.) were further described as helpful. Although the use of activities in FT sessions is common, it is not a widely researched area. Existing literature on the topic relates mostly to engaging children in sessions (Sori 2006). While there are many books and articles providing a plethora of structured therapeutic activities, responsible use of these is often not sufficiently emphasized (Brown 2018; Yalom and Leszcz 2005); thus, therapists have a responsibility to ensure safety from any kind of harm in practice.

### 8.3 Intervention Delivery

A collaborative delivery approach where the family is positioned as the expert in their lives, with respect for their needs, goals, and preferences correlated with engagement and confidence in the process. Contrary to some research findings that suggested the “therapist‐as‐expert” stance was preferable and that collaborative approaches were “merely a conceptual idea” (Bischoff and McBride 1996, p. 125), there is much recent research that supports a collaborative delivery (Gehart 2018). This suggests a shift in the power dynamic and sense of ownership in the last two decades and supports common factors theories that the client's role in therapy is a key factor of change (Wampold 2015). Findings suggest that families lost confidence and disengaged when their preferences were not sufficiently considered and the intervention was not relevant to their needs and goals, obscuring their view of how they might benefit. This supports the previous findings of Chenail et al. (2012). Moreover, a strengths‐based view that affirmed and reinforced family strengths and resources, encouraging and motivating families, was found helpful. This also supports existing research (Sackett and Cook 2022).

These findings suggest that a collaborative, feedback‐informed approach is preferred, requiring counselors to keep current client goals in view and continuously check with clients about what is and is not working (Holyoak et al. 2021). They further point to an important implication for practitioners regarding positioning the family as an “equal,” especially when delivering interventions where practitioners might automatically be seen as the expert; for example, where the family has been referred or manualized treatments such as those in this review. Further, families who have experienced marginalization and social oppression are more likely to engage in therapy if they are offered a collaborative, equal style of intervention (Fraenkel 2006). In the Aotearoa/New Zealand context, this is an important consideration for practitioners who work with family and community systems that have been impacted by colonization, such as Māori families. While keeping the focus on the family's resilience and potential is necessary, it is important that their concerns and painful experiences are not minimized; thus, practitioners need to be mindful of balancing these elements.

Families also found it helpful when practitioners imparted knowledge and information using age‐appropriate and jargon‐free language. Likewise, skills coaching in tools such as assertive communication, parenting, and emotional regulation was valued. The implication for counselors is incorporating knowledge‐sharing into sessions (Sackett and Cook 2022), which further enhances the equal nature of the relationship.

Finally, practical aspects of therapy were discussed. While for some families home‐based sessions were the reason for perseverance, some young people found them distracting. They further found themselves with no say in scheduling sessions, creating resentment and disengagement. Although for many counselors home‐based sessions might not be possible, flexibility in conducting sessions where the family feels comfortable and safe should be a conversation (Synder and McCollum 1999). Further, some families cited concern regarding how therapy ended. These aspects require collaborative consideration, endeavoring to ensure that the safe and trusting bond that takes time to establish does not end abruptly but is safely tapered (Lebow and Gurman 1995).

### 8.4 Family Engagement with the Process

While a secure therapeutic relationship enabled clients to be themselves and share their stories, when they were asked to do this before it was established, clients (especially young people) experienced distress (e.g., Collyer et al. 2021). Implications for practice are that counselors observe indications of client comfort and safety and gauge how they feel about discussing difficult topics and painful experiences, respecting differences in the time and number of sessions it takes for individuals to feel safe to do so.

Families noted the helpfulness of attending sessions as a family. This supports Chenail et al.'s (2012) findings. They further identified the preference for a combination of whole‐family, extended‐family, family sub‐groups (e.g., mother and child, siblings, etc.), and individual sessions; counselors must acknowledge this requirement and endeavor to provide these options when possible. They must also be especially careful when younger children are present in sessions to ensure their voices are heard and valued while gauging their stress levels; this is supported by existing literature (Stith et al. 2022).

### 8.5 Strengths, Limitations, and Recommendations for Future Research

A notable strength of this review is its synthesis of recent literature, with most studies published in the last several years, increasing the relevance of results to current practice. The included literature is mostly of good quality, providing reliable data. A recommendation for the next wave of systematic reviews would be to include findings of both qualitative and quantitative studies.

The review has several limitations. First, a significant limitation of this study is its shortage of synthesizable data on unhelpful components of family sessions as some studies only reported positive findings (what families found helpful). This raises questions of researcher bias. Where the researcher–participant relationship was not clear, impartiality and candidness of participant views might have been impacted; for example, where the FT process was at the initial stages and continuing. Additionally, the views of families who dropped out were limited. It is imperative that future research focuses on giving voice to FT clients without guiding them toward seemingly biased outcomes.

Second, although the identified client was an adolescent/young person in all the selected studies, fewer of them (by 24%) participated in the studies. Despite this, young people identified unhelpful experiences at twice the frequency as the parents (20% vs. 11% of total responses). Future research should explore how this perception of being identified as the “problem family member” impacts young people's views and experiences of the FT process. Additionally, in two studies family members were interviewed together, which leads to questions about the influence parents' presence had on their children's openness. The findings of this review applied to all family members even though more parents participated in the studies. It is important to understand children and young people's genuine views of FT; future research should recruit more children and adolescents as research participants, and research questions should be formulated to ensure that their distinct experiences of FT are better understood. It should also aim to uncover best practices for including young children in FT as well as engaging older children and young people. These implications support existing literature (Helimäki et al. 2022b; Sori 2006).

Third, the review is limited to English‐language articles published in peer‐reviewed journals, introducing publication bias. Further, while studies spanned several countries (including countries where the primary language is not English) and included participants of several ethnicities, all but one study was based in Western countries, limiting generalizability and applicability to non‐Western settings. While it is expected that the findings may generalize somewhat to the Aotearoa/New Zealand context, it cannot be assumed that they apply to indigenous/Māori families.

Fourth, participating families are not representative of all families who might present for FT. Further studies with diverse types of families such as LGBTQ+ families, blended families, single‐parent families, interracial families, and families with diverse socioeconomic backgrounds should be carried out to determine if findings can be generalized.

Finally, one researcher took primary responsibility for conducting each step of the systematic review. This is less desirable than two researchers independently performing each step; however, the second and third authors critically examined the process and outcomes.

## 9 Conclusion

This qualitative systematic review analyzed recent literature on client experiences of FT. Despite variations in FT models, countries, participant demographics, and settings, comparable perceptions of what families found helpful and what they found unhelpful were reported. The most frequently identified helpful component of FT was the impact of a safe and supportive therapeutic relationship on the therapeutic process. Therapists' characteristics and qualities that enabled them to connect with the family system and their nonjudgmental, respectful, and culturally sensitive approaches were found to significantly contribute to this alliance, pointing to the importance of personal and professional development of practitioners with highly effective relational skills and self‐awareness. Second, the therapeutic techniques and skills that practitioners used during sessions were identified as helpful. Therapists' interviewing skills, how they facilitated perspective shifts, and the experiential activities incorporated into sessions to facilitate communication, emotional expression, and insight were found helpful, suggesting the significance of counselor education that includes skill‐based components, which incorporate various FT techniques. Third, families valued having influence over the content, format, and process of therapy; having their strengths and resources reinforced; and receiving information and strategies—highlighting the importance of collaborative therapeutic processes. Finally, sharing their concerns openly as a family and the positive results that they saw after the initial sessions were found helpful. Families also identified several unhelpful aspects of FT sessions, such as being expected to share before they felt comfortable; when the process, format, content, or proposed solutions did not address their needs; when they did not observe sufficient progress in the early stages; and when therapists took sides or were unable to manage conflict during sessions.

## Endnotes

1 Selected studies will be referred to in superscript, as per the study numbers in Table 2, to improve readability.
